# Mycofactocin-associated mycobacterial dehydrogenases with non-exchangeable NAD cofactors

**DOI:** 10.1038/srep41074

**Published:** 2017-01-25

**Authors:** Daniel H. Haft, Phillip G. Pierce, Stephen J. Mayclin, Amy Sullivan, Anna S. Gardberg, Jan Abendroth, Darren W. Begley, Isabelle Q. Phan, Bart L. Staker, Peter J. Myler, Vasilios M. Marathias, Donald D. Lorimer, Thomas E. Edwards

**Affiliations:** 1 National Institutes of Health (NIH), Bethesda, MD 20892, USA; 2 Seattle Structural Genomics Center for Infectious Disease (SSGCID), Seattle, WA 98109, USA; 3 Beryllium Discovery Corp., 7869 NE Day Road West, Bainbridge Island, WA 98110, USA; 4 Center for Infectious Disease Research (formerly Seattle Biomedical Research Institute), 307 Westlake Avenue North, Seattle WA 98109, USA; 5 University of Washington, Department of Medical Education and Biomedical Informatics & Department of Global Health, Seattle WA 98195, USA

## Abstract

During human infection, *Mycobacterium tuberculosis* (*Mtb*) survives the normally bacteriocidal phagosome of macrophages. *Mtb* and related species may be able to combat this harsh acidic environment which contains reactive oxygen species due to the mycobacterial genomes encoding a large number of dehydrogenases. Typically, dehydrogenase cofactor binding sites are open to solvent, which allows NAD/NADH exchange to support multiple turnover. Interestingly, mycobacterial short chain dehydrogenases/reductases (SDRs) within family TIGR03971 contain an insertion at the NAD binding site. Here we present crystal structures of 9 mycobacterial SDRs in which the insertion buries the NAD cofactor except for a small portion of the nicotinamide ring. Line broadening and STD-NMR experiments did not show NAD or NADH exchange on the NMR timescale. STD-NMR demonstrated binding of the potential substrate carveol, the potential product carvone, the inhibitor tricyclazol, and an external redox partner 2,6-dichloroindophenol (DCIP). Therefore, these SDRs appear to contain a non-exchangeable NAD cofactor and may rely on an external redox partner, rather than cofactor exchange, for multiple turnover. Incidentally, these genes always appear in conjunction with the *mftA* gene, which encodes the short peptide MftA, and with other genes proposed to convert MftA into the external redox partner mycofactocin.

Redox enzymes that bind a single NAD cofactor can act in several conceptually different ways. In scheme A, the enzyme can oxidize a substrate by reducing NAD+ to NADH, and then allow both the product and the cofactor to diffuse away. Alcohol dehydrogenase is an example of such an enzyme^1^. The NADH it releases may be re-oxidized to NAD+ elsewhere to allow the enzyme to perform multiple turnovers. In scheme B, the NAD cofactor remains tightly bound, and no net redox change to the substrate occurs. The substrate is reduced, then re-oxidized, such that the substrate is converted from one isomer to another without a net change in oxidation state. UDP-galactose-4-epimerase is an example of such an enzyme^2^. In scheme C, the NAD cofactor remains tightly bound and does not dissociate from the enzyme after the physiological substrate undergoes a net redox change; instead electron transfer occurs between a bound NAD+ and free NADH. In scheme D, the NAD is tightly bound and cannot exchange, the enzyme catalyzes a steady flux for some substrate into its product, but the enzyme achieves multiple turnovers by alternating between oxidation of its primary substrate with reduction of an unrelated molecule called a co-substrate. As an example, PdxB oxidizes 4-phospho-d-erythronate to 2-oxo-3-hydroxy-4-phosphobutanoate, but afterwards it reduces a co-substrate such as α-ketoglutarate, oxaloacetate, or pyruvate to regenerate NAD+ from its NADH^3^. It achieves multiple turnovers, despite its non-exchangeable cofactor, by tying net oxidation of one substrate to net reduction of another.

In scheme E, oxidoreductases with non-exchangeable NAD+/NADH cofactors rely on an external redox system to mediate recycling of NAD+ to NADH while it remains in the enzyme’s cofactor-binding site. In contrast to universal cofactors such as NAD+ and NADP, such a system might rely on a specialized redox carrier whose biosynthesis shows a limited and sporadic distribution among bacteria whose genomes have been sequenced. An intriguing example of such a system is pyrroloquinoline quinone (PQQ). In the PQQ biosynthetic pathway, a radical SAM enzyme (PqqE) participates in modification of a ribosomally translated short peptide (PqqA) as a key step in the biosynthesis of a redox carrier on which whole families of enzymes depend^4^.

Based off genomic analysis we recently identified a widely distributed set of three uncharacterized genes, found in *Mycobacterium tuberculosis* and dozens of other species, and proposed that this system is responsible for the biosynthesis of a peptide-derived natural product we named mycofactocin which is similar in function to PQQ^5^. The three genes proposed to be involved in mycofactocin biosynthesis always appear together or not at all: mftA encodes a short peptide with a C-terminal sequence IDGXCGVY, mftB encodes a peptide chaperone, and mftC encodes a radical S-adenosyl methionine enzyme (rSAM).

Recently, the recombinant expression and characterization of MftA, MftB, and MftC have been reported^6^,^7^. The chaperone MftB was reported to bind the mycofactocin precursor MftA with an affinity of approximately 120 nM and the rSAM MftC with an affinity of approximately 2 μM. In addition, MftC was shown to catalyze the decarboxylation of the C-terminal tyrosine of MftA in the presence of MftB, presumably the first step in the biosynthetic pathway of mycofactocin.

Other genes often appear in close proximity to the mftA, mftB, and mftC genes such as mftD which encodes a heme/flavin oxidoreductase, a glycosyltransferase, a creatininase, and numerous short chain dehydrogenases/reductases (SDRs) of the protein family TIGR03971 previously identified as a distinctive clade of PF00106^5^. These SDRs occur only in species with the genomic markers of mycofactocin biosynthesis, and often are encoded by genes neighboring those markers. Interestingly, a distinctive feature of the SDRs from TIGR03971 is an insertion in the primary sequence near the expected NAD binding site that is absent in other oxidoreductases. By and large, none of these SDR genes have been characterized beyond genomic analysis. In a rare example, a homologous SDR from *Rhodococcus erythropolis* DCL14 has been characterized as a stereoselective carveol dehydrogenase which has a permanently bound, non-exchangeable NAD^8^. Investigators found that they needed to use artificial electron donors or acceptors such as methanol:N,N-dimethyl-4-nitrosoaniline (NDMA) or 2,6-dichloroindophenol (DCIP) to recharge NAD to its prior state after every reaction to enable the enzyme to cycle through multiple turnovers. Hence, under scheme E, certain oxidoreductases use a specialized electron transfer machinery to recharge captive NADH rather than using an artificial carrier such as NDMA or DCIP. Analogy to PQQ biosynthesis suggests that MftA is the precursor of a peptide-derived redox carrier central to the proposed redox transfer system, and recent evidence that modification occurs at the C-terminal tyrosine helps support that hypothesis.

Here we present a series of TIGR03971 SDR crystal structures from several mycobacterial species which contain an insertion in the primary sequence at the NAD cofactor binding site. These crystal structures demonstrate that the inserted protein residues almost completely cover the NAD cofactor, only leaving part of the nicotinamide ring exposed to solvent. NMR experiments did not show exchange of the NAD cofactor, whereas binding was demonstrated for a number of small molecules which could be potential substrates, products, inhibitors, or artificial redox partners. Experimental results are consistent with the notion that the SDR family enzymes linked by comparative genomics studies to mycofactocin do indeed keep their NAD tightly bound beneath a protein structure that may interact with a specialized adaptor machinery for redox exchange. The mature product of the mycofactocin biosynthesis system may be the critical redox carrier of that adaptor machinery.

## Results

### Genome analysis of mycobacterial SDRs

**Figure 1:**
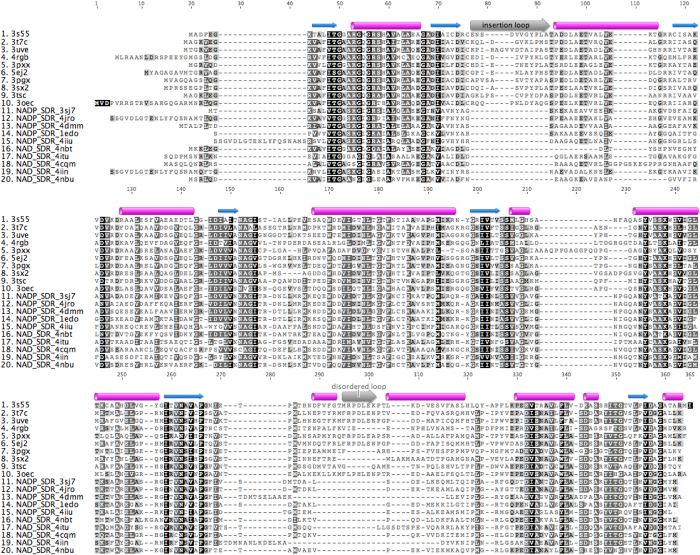
Genome structure and sequence analysis of mycobacterial SDRs. A number of SDRs containing an insertion at the NAD binding site appear in mycobacterial organisms. These SDRs always appear in conjunction with a number of genes that appear to be involved in the biosynthesis of the proposed redox partner mycofactocin. Multiple sequence alignment of the ten crystal structures determined here in comparison with 10 other oxidoreductases of known structure which lack the insertion in the primary sequence. The PDB code for each structure is listed. The structures described here all contain NAD (sequences 1–9) except for 3OEC (sequence 10), whereas for the structures described elsewhere 5 representative examples of NADP (sequences 11–15) or NAD (sequences 16–20) bound structures were selected and the ligand identity is also listed. The magenta cylinders indicate α helices, whereas the blue arrows indicate β strands. The insertion loop is shown as a gray arrow and labeled, and the loop region that is often disordered is also shown as a gray arrow and labeled.

Members of TIGR03971 appear in gene clusters near mycofactocin system protein genes mftA, mftB, and mftC^5^,^6^,^7^. For example, in *M. paratuberculosis* a putative carveol dehydrogenase (MAP_RS21280, UniProt ID Q73SC8, SSGCID target ID MypaA.01326.b) is encoded near genes for the mycofactocin precursor MftA (MAP_RS21310), the mycofactocin modification chaperone MftB (MAP_RS21315), the mycofactocin radical SAM maturase MftC (MAP_RS21320), the mycofactocin system heme/Flavin oxidoreductase MftD (MAP_RS21325), and the mycofactocin system creatininase family protein (MAP_RS21330). The presence of an insertion in the primary sequence near the proposed NAD-binding region is conserved across members of TIGR03971, but absent in other NAD- or NADP-dependent oxidoreductases with known structure (Fig. 1). Based off genomic analysis, 69 mycobacterial dehydrogenases from TIGR03971 with an insertion near the proposed cofactor binding site were entered into the Seattle Structural Genomics Center for Infectious Disease (SSGCID) structure determination pipeline^9^. Of these, 42 targets (61%) progressed to successful protein production. Each target was selected on the basis of sequence similarity to either Mtb Rv0687 (100% sequence identity to Mybo.01326.a) or Rv2750 (MytuD.01326.a). Originally, these two Mtb targets progressed through the pipeline to purified protein, but failed to yield suitable diffraction quality crystals.

### Structural analysis of mycobacterial SDRs

**Figure 2:**
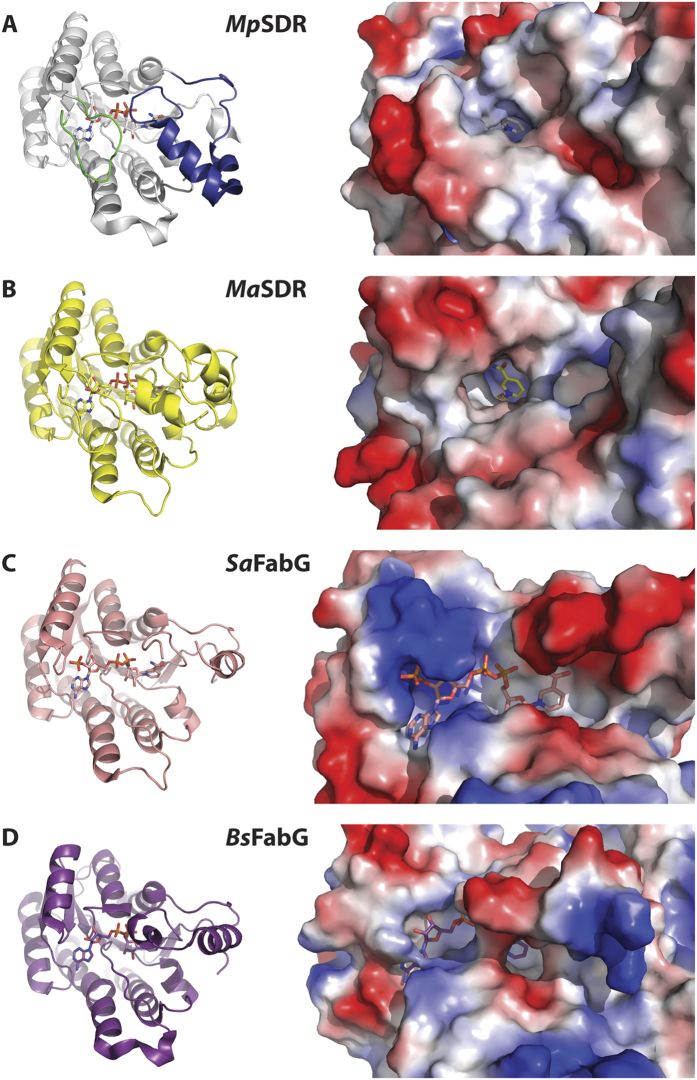
Structural analysis of mycobacterial SDR NAD binding sites. (**A**) Carveol dehydrogenase from *M. paratuberculosis* (MypaA.01326.b) bound to NAD (PDB ID 3PGX). The insertion loop which cover the NAD is shown in green and the loop often disordered in other SDR crystal structure is show in blue (left) and surface rendering showing the available solvent channel with hydrophobic regions shown in white, electropositive regions shown in blue and electronegative regions shown in red (right). (**B**) SDR from *M. abscessus* (MyabA.01326.f) bound to NAD (PDB ID 3S55). (**C**) FabG oxidoreductase from *Staphylococcus aureus* bound to NADPH (PDB ID 3SJ7)^23^. (**D**) FabG oxidoreductase from *Bacillus sp. SG-1* bound to NAD (PDB ID 4NBU)^24^. This figure is intended to be representative examples of crystal structures of SDRs with the insertion loop determined here (panels A, B) with those bound to a more open NAD/NADP (panel C) and those bound to a more closed NAD/NADP (panel D); see Table 2 for calculated solvent accessibility of these and other structures.

We determined crystal structures for 10 different mycobacterial SDRs (Tables 1, 2 and 3). These ranged in sequence identity to Rv0687 from 39–81% and from 41–77% for Rv2750. Each of these SDRs contain an insertion in the protein sequence at the cofactor binding site (Fig. 1). With the exception of the *M. thermoresistibile* target which did not contain NAD, all other mycobacterial SDR target crystal structures contained NAD, which appears to have co-purified with the protein after expression in *E. coli*. For each of these crystal structures, the adenosine mononucleotide portion of the cofactor binding site is enveloped by the inserted residues and only a portion of the nicotinamide ring appears open to solvent (see representative examples in Fig. 2A and B).

In addition to the insertion loop which covers the adenosine portion of the cofactor, a second loop which typically contains two α-helices covers much of nicotinamide mononucleotide portion of the cofactor (Fig. 2A). As observed in other structures (see representative examples in Fig. 2C and D), this loop can adopt a number of different conformations and is often disordered. In contrast, this loop is ordered in each of the mycobacterial SDR crystal structures presented here. The combination of the insertion loop which covers the adenosine portion of the cofactor and the ordering of the second loop which covers the nicotinamide mononucleotide portion of the cofactor results in a significant decrease in the solvent accessible surface area of the cofactor in the mycobacterial SDRs, relative to other NAD- and NADP-dependent dehydrogenases (Table 4). Only a small portion of the nicotinamide ring appears available to solvent in each of these cases, indicating that the NAD cofactor is tightly bound and potentially non-exchangeable. The structures presented here have a cofactor solvent accessible surface area of approximately 18 Å^2^, whereas other NAD- or NADP-bound dehydrogenases have a cofactor solvent accessible surface area of 82–89 Å^2^ (Table 4).

### Exchangeability of cofactor NAD/NADH by line broadening NMR and STD-NMR

A number of NMR experiments were performed to test exchangeability of the NAD cofactor. NMR line broadening experiments rely on differences in tumbling times between free and bound ligand in solution to detect protein-ligand interactions^10^,^11^. In the absence of protein, small molecule tumbling time is relatively fast, resulting in sharp peaks; upon binding to a protein, the tumbling time of the complexed small molecule is slowed, resulting in less intense and broader resonance peaks. Thus, one can compare the peaks of a particular ligand in the absence or presence of protein at specific concentrations to identify and characterize binding interactions. Line broadening was examined for both NAD and NADH in the absence and presence of two *M. avium* carveol dehydrogenases (MyavA.01326.d and MyavA.01326.l). No line broadening was observed for either NAD or NADH in the absence or presence of either protein over a concentration of 0.1–2.0 mM. Thus, neither NAD nor NADH appears to bind to the protein over the course of the experiment. The presence of a previously-bound, tightly-binding cofactor in the binding pocket of either protein could prevent additional NAD or NADH added to solution from undergoing ligand exchange. Thus, the lack of a line broadening effect is consistent with non-exchangeable cofactor binding.

The saturation transfer difference NMR (STD-NMR) experiment is designed to detect compounds which are capable of rapid to intermediate ligand exchange with a target protein, and is well-suited for the identification of weak- to medium-strength binding ligands^12^,^13^. Typically, STD-NMR saturation is not observed for proton resonances of non-binding or very tight binding small molecules (K_D_ < 50 nM); the slow off-rate does not allow sufficient numbers of molecules to be detected over the course of the experiment. STD-NMR experiments were performed on both NAD and NADH in the absence and presence of two *M. avium* carveol dehydrogenases (MyavA.01326.d and MyavA.01326.l). No significant STD-NMR signal was observed for either NAD or NADH. Given that these enzyme samples (MyavA.01326.d and MyavA.01326.l) both generated crystal structures with NAD present without supplementation, the non-binding result by STD-NMR is most likely reflective of non-exchangeable binding, consistent with the results observed by line-broadening NMR.

### Binding of potential substrates, products, inhibitors and redox partners by STD NMR

The binding of potential substrates, products, inhibitors, and redox partners to two *M. avium* carveol dehydrogenases (MyavA.01326.d and MyavA.01326.l) was examined by STD NMR. No binding signal was observed for (−)-carveol, (R)-(−)-carvone, (S)-(+)-carvone, or tricyclazol in the presence of NAD and/or NADH in the absence of protein. Both of the redox partner compounds NDMA (N,N-dimethyl-4-nitrosoanaline) and 2,5-dichloroindophenol appear to enhance oxidation of NADH to NAD over the course of 8 days, relative to NADH alone in solution. However, none of the non-exchangeable protons of either NDMA or 2,5-dichloroindophenol experiences peak height or chemical shift changes over the same duration, suggesting these molecules remain intact and do not chemically react with either NAD or NADH directly.

For the *M. avium* carveol dehydrogenase MyavA.01326.d, no significant (>10%) STD NMR effect was observed for (−)-carveol, (R)-(−) carvone, (S)-(+)-carvone or NDMA (Table 5). Binding was observed for tricyclazol and DCIP, with the latter generating twice the STD binding signal as the former. STD NMR binding of each compound was monitored after spiking samples with either NAD or NADH to mimic the effect of soluble cofactor. For the potential redox partner DCIP, the %STD NMR signal was initially diminished upon spiking with NADH but not NAD, while the weak binding signal of tricyclazol experienced no change with either cofactor added to the sample. The STD binding signal of (−) carveol experienced a near 4% increase upon addition of NAD, relative to binding in the absence of cofactor. All spiked samples were retested by STD NMR after a 7-day incubation period. The STD NMR binding signal for DCIP returned to pre-spike levels, and the STD NMR binding of NMDA increase after 7 days of incubation. As was observed for NDMA and DCIP in the absence of protein, we see partial conversion of NADH to NAD over time in the presence of protein.

**Figure 3:**
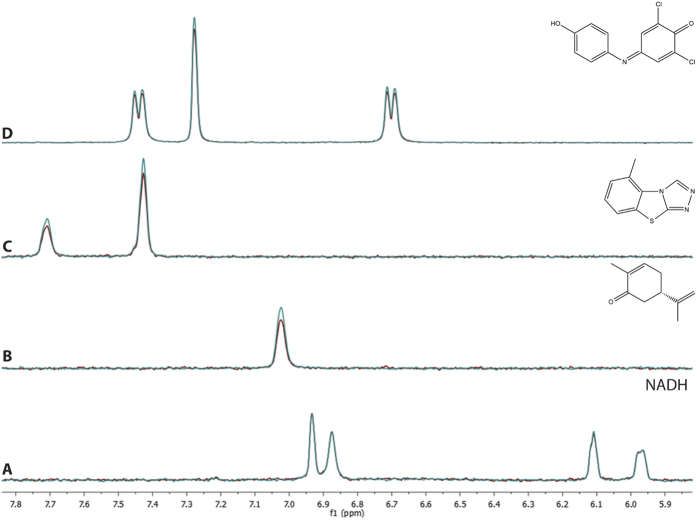
NMR spectroscopic analysis of mycobacterial SDR ligand binding. Reference (blue) and saturation (red) STD NMR spectra overlaid for a *M. avium* SDR (SSGCID ID MyavA.01326.l) in the presence of (**A**) cofactor NADH (2.1% max STD signal), (**B**) potential product (S)-(+)-carvone (20.5% max STD NMR signal), (**C**) potential inhibitor tricyclazol (19.0% max STD NMR signal), and (**D**) redox partner DCIP (10.2% max STD NMR signal). Although NADH appears to be a non-binder under these conditions, the other compounds all show clear effects of binding.

STD-NMR binding was then examined for the *M. avium* carveol dehydrogenase MyavA.01326.l (Fig. 3 and Table 6). Weak but clear STD NMR binding signal was observed for carveol and DCIP, with stronger saturation signal for (R)-(−)-carvone, (S)-(+)-carvone and tricyclazol. No significant binding effect was observed for NMDA. Spiking NAD into the sample reduced the the STD NMR binding signal for DCIP. As was observed with MyavA.01326.d, the STD NMR binding signal of DCP returned to pre-spike levels after 7 days of incubation. As was observed for NDMA and DCIP in the presence of MyavA.01326.d, partial conversion of NADH to NAD was observed after 7 day incubation in the presence of MyavA.01326.l.

## Discussion

The presence of a large number of dehydrogenases present in mycobacterial genomes may enhance the chances for survival in the harsh redox conditions present in macrophages. Some of these dehydrogenases may have elaborate control mechanisms beyond those currently understood to function in human host cells. Previously, we proposed the existence of an external redox partner specific to mycobacteria and related species called mycofactocin^5^. A series of genes always appear in close proximity to one another within mycobacteria and other closely related organisms. These include an open reading frame encoding the small peptide precursor of mycofactocin (MftA, TIGR03969), a carrier protein (MftB, TIGR03967), a radical SAM protein (MftC, TIGR03962), a glycosyltransferase (TIGR03965), and one or more redox proteins. Interactions of MftA, MftB, MftC as well as the decarboxylation of MftA by the MftC/MftB complex have been reported recently^6^,^7^. In addition, most of these gene clusters also include a short chain dehydrogenase/reductase (SDR) from TIGR03971, the protein family focus of the current research. These SDRs are readily distinguishable from other SDRs on the protein level because of a large insertion in the primary sequence (Fig. 1). This insertion warranted detailed study since it is located near the adenosine binding region of the cofactor NAD (or NADP) in other SDRs with known structure. To characterize the importance of these additional protein residues, we determined the crystal structures of a number of mycobacterial SDRs containing this insertion. In these structures, the insertion loop covers the adenosine portion of the NAD cofactor. Concomitantly, another loop which is present but often disordered in other SDR crystal structures is ordered for the TIGR03971 family SDR protein structures and covers much of the nicotinamide mononucleotide portion of the NAD cofactor (Fig. 2). Taken together, these ordered loops in mycobacterial SDRs significantly cover the bound NAD cofactor, resulting in a solvent accessible surface area for the cofactor of only 18 Å^2^ (Table 4). This solvent accessible surface area is significantly diminished in comparison with other representative NAD-dependent SDRs which have an average solvent accessible surface area of 89 Å^2^. Similarly, representative NADP-dependent SDRs have an average solvent accessible surface area of 82 Å^2^. It should be noted that the calculated surface areas are representative of a select number of cofactor-bound SDR protein structures in the Protein Data Bank. Interestingly, BphB from *Pandoraea pnomenusa* also has a highly buried NAD molecule, but it accomplishes this not through an insertion as shown for these mycobacterial SDRs, but rather through an extension of the loop which normally only buries the adenosine portion of NAD^14^. This effectively creates a long cavity in which small molecules (i.e. potential substrates) can bind.

Given the encapsulation of the NAD cofactor by the two loops in mycobacterial SDRs, a large conformational change would be required to release the NADH cofactor after a redox reaction had occurred. Alternatively, these structural elements may not allow for exchange of bound NAD/NADH with free NAD/NADH. If these enzymes used a non-exchangeable NAD, they would either be single turnover enzymes or would require an external redox partner. Other oxidoreductases which utilize external redox partners have been characterized enzymatically^8^, but no structural information exists for these kinds of SDRs. We know from the crystal structures presented here that these protein samples indeed contain bound NAD. To test whether or not the bound NAD was exchangeable, we performed a number of line broadening and STD-NMR ligand-observe studies, and did not observe exchange of NAD/NADH during the timescale of the NMR experiments. In contrast, we were able to observe binding of potential enzymatic substrates such as carveol, potential products such as carvone, potential inhibitors such as tricyclazol, and external artificial redox partners such as DCIP.

The structural and NMR binding data are consistent with the hypothesis that the mycobacterial SDRs in TIGR03971 utilize a non-exchangeable NAD and require an external redox partner. Given the proximity of these TIGR03971 mycobacterial SDR genes to a cluster of enzymes which have been hypothesized to be involved in the biosynthesis of mycofactocin^5^ and that the initial decarboxylation of MftA by the MftC/MftB complex has been characterized enzymatically^6^,^7^, we speculate that these SDRs may use mycofactocin as an external redox partner. However, if these enzymes use an external redox partner such as mycofactocin, one might expect the binding sites to be similar for all of these enzymes, despite different catalytic functions and substrates. Interestingly, while the presence of the insertion in the primary sequence is conserved across these mycobacterial SDRs, the actual sequence does not appear to be conserved (Fig. 1). Thus, this observation may not be consistent with the hypothesis of mycofactocin as an external redox partner if mycofactocin requires highly specific interactions with the SDRs. However, we note that although the C-terminus of the mycofactocin precursor MftA is highly conserved, the length (30–80 amino acids) and the remaining sequence of MftA is not well conserved^7^, and differences in specific MftA sequences may be paired with differences in the SDR insertion sequences as well. Nevertheless, these SDRs always appear in close proximity to the TIGR03969 gene which encodes mycofactin (MftA) and other proteins (the carrier protein MftB TIGR03967, a radical SAM protein MftC TIGR03962, and a glycosyltransferase TIGR03965). Additional genomic, biochemical, and structural analysis is necessary to understand the importance of these proteins in the biosynthesis of mycofactocin and the subsequent redox pathways.

## Methods

### Overexpression and purification

All proteins were overexpressed in *E. coli* with an N-terminal His 3C protease cleavage fusion using protocols described previously^15^,^16^. Briefly, the proteins were purified by nickel affinity chromatography followed by optional cleavage and removal of the fusion tag with 3C protease and a subtractive nickel column and a final size exclusion chromatography (SEC) step. The affinity tag was not removed for MyavA.01326.d, MyavA.01326.q, and MythA.01326.c, but it was removed for all of the other samples. The final samples were stored at −80 °C in 25 mM Tris pH 7.0, 500 mM NaCl, 5% v/v glycerol, 2 mM DTT and 0.025% w/v NaN_3_ until used in crystallization experiments.

### Crystallization

Crystals were grown using the sitting drop vapor diffusion method at 289 K with 0.4 μL protein and 0.4 μL precipitant equilibrated against 80 μL of reservoir. Crystals of MyabA.01326.f were obtained at 27.3 mg/mL against the JCSG+ screen condition D10 which contains 0.2 M calcium acetate, 0.1 M sodium cacodylate pH 6.5, and 40% w/v PEG 300; crystals were harvested with a direct cryo-protectant. Crystals of MyavA.01326.d were obtained at 43.4 mg/mL against the JCSG+ screen condition H3 which contains 25% w/v PEG 3350, 0.1 M BisTris pH 5.5; crystals were harvested with 20% v/v ethylene glycol as cryo-protectant. Crystals of MyavA.01326.e were obtained at 48 mg/mL against a focus screen based off the JCSG+ screen condition C6 which contained 42.5% w/v PEG 300, 0.1 M phosphate citrate pH 4.62 crystals were harvested with a direct cryo-protectant. Crystals of MyavA.01326.g were obtained at 20 mg/mL supplemented with 2.5 mM NAD against MCSG1 condition C12 which contains 25% w/v PEG 3350 and 0.1 M BisTris pH 6.5; crystals were harvested with 15% v/v ethylene glycol as cryo-protectant. MyavA.01326.i crystallized at 36.4 mg/mL against the JCSG+ screen condition A5 which contains 20% w/v PEG 3350 and 0.2 M magnesium formate; crystals were harvested with 25% v/v ethylene glycol as cryo-protectant. Crystals of MyavA.01326.q were obtained at 26.9 mg/mL against the JCSG+ screen condition A11 which contains 50% v/v MPD, 0.2 M ammonium dihydrogen phosphate, 0.1 M Tris pH 8.5; crystals were harvested with 15% v/v ethylene glycol as cryo-protectant. Crystals of MypaA.01326.b were obtained at 23.8 mg/mL against the JCSG+ screen conditions H11 which contains 25% w/v PEG 3350, 0.2 M magnesium chloride, 0.1 M BisTris pH 5.5; crystals were harvested with 25% v/v ethylene glycol as cryo-protectant. Crystals of MypaA.01326.d were obtained at 23 mg/mL against a focus screen based off the JCSG+ screen condition D5 which contained 70% v/v MPD and 0.1 M Hepes pH 6.5; crystals were harvested with a direct cryo-protectant. Crystals of MypaA.01326.g were obtained at 27.6 mg/mL against the JCSG+ screen condition D5 which contained 70% v/v MPD and 0.1 M Hepes pH 7.5; crystals were harvested with a direct cryo-protectant. Crystals of MythA.01326.c were obtained at 29.5 mg/mL against the JCSG+ screen condition E11 which contains 14.4% w/v PEG 8000, 0.16 M calcium acetate, 80 mM sodium cacodylate pH 6.5 and 20% v/v glycerol; crystals were harvested with a direct cryo-protectant.

### X-ray data collection and structure determination

X-ray data were collected either in house on a Rigaku FR-E+ SuperBright X-ray generator with Saturn 944+ detectors or at synchrotron radiation (Tables 2 and 3). Data were reduced with XDS^17^ using a single crystal for each target. All structures were solved by molecular replacement in Phaser^18^. The final models (Tables 2 and 3) were obtained after iterative refinement in Refmac5^19^ or Phenix^20^ with manual model building in Coot^21^, and validation in MolProbity^22^.

### NMR analysis

All experimental NMR data were obtained on a Varian 500 MHz. Two enzymes annotated as carveol dehydrogenases from *M. avium* (MyavA.01326.d and MyavA.01326.l) were dialyzed into an NMR specific buffer (150 mM NaCl, 20 mM potassium phosphate, 0.1 mM NaN_3_, 10% D_2_O pH 7.0). Line broadening experiments were performed on both MyavA.01326.d and MyavA.01326.l with the protein at 20 μM concentration and the ligands at 250 μM with a constant 5% v/v DMSO-d_6_ in the above NMR buffer. Line broadening of NAD and NADH was examined in the absence of protein as a control and in the presence of protein at concentrations of 0.1, 0.25, 0.5, 1.0 and 2.0 mM ligand. STD NMR experiments were performed on both MyavA.01326.d and MyavA.01326.l with the same conditions as the line broadening experiments for singleton STD NMR. STD NMR spike experiments were done with addition of NAD/NADH to a final concentration of 250 μM. As control experiments, all compounds were tested for stability in the NMR buffer over a period of 8 days. The potential inhibitor N,N,N′,N′-tetramethyl-p-phenylenediamine (TMPD) was not stable in the NMR buffer over 8 days and thus was not selected for additional experiments. Each compound was also tested for direct irradiation by STD-NMR in the absence of protein; false binding signals were not observed for any of compound in these experiments. The reported %STD NMR binding signal is the peak height difference (reference – saturation) for the single strongest proton resonance of the compound.

### Data Availability

The expression plasmids and surplus protein samples can be obtained from either the contact authors or through www.ssgcid.org and the raw X-ray diffraction data can be obtained through www.csgid.org.

## Additional Information

**Accession codes:** Atomic coordinates for the reported structures have been deposited with the Protein Data Bank under accession codes 3OEC, 3PGX, 3PXX, 3S55, 3SX2, 3T7C, 3TSC, 3UVE, 5EJ2, and 4RGB.

**How to cite this article**: Haft, D. H. *et al*. Mycofactocin-associated mycobacterial dehydrogenases with non-exchangeable NAD cofactors. *Sci. Rep.* **7**, 41074; doi: 10.1038/srep41074 (2017).

**Publisher's note:** Springer Nature remains neutral with regard to jurisdictional claims in published maps and institutional affiliations.

## Figures and Tables

**Table 1:** Crystal structures of various mycobacterial dehydrogenases containing the identified insert.

Annotation	Species	UniProt ID	SSGCID ID	Cofactor	PDB ID	Resolution	Mtb gene
Putative short-chain dehydrogenase	*M. abscessus*	B1MLR7	MyabA.01326.f	NAD	3S55	2.10 Å	Rv0687
Carveol dehydrogenase	*M. avium*	A0QCJ8	MyavA.01326.d	NAD	3T7C	1.95 Å	Rv2750
Carveol dehydrogenase	*M. avium*	A0QB72	MyavA.01326.e	NAD	3UVE	1.55 Å	Rv2750
Putative carveol dehydrogenase	*M. avium*	A0QDP5	MyavA.01326.g	NAD	4RGB	1.95 Å	Rv2750
Carveol dehydrogenase	*M. avium*	A0QFV1	MyavA.01326.l	NAD	3PXX	2.0 Å	Rv2750
Carveol dehydrogenase	*M. avium*	A0QGY1	MyavA.01326.q	NAD	5EJ2	2.15 Å	Rv2750
Putative carveol dehydrogenase	*M. paratuberculosis*	Q73SC8	MypaA.01326.b	NAD	3PGX	1.85 Å	Rv0687
Putative 3-ketoacyl-ACP reductase	*M. paratuberculosis*	Q73W00	MypaA.01326.d	NAD	3SX2	1.50 Å	Rv2750
Short chain dehydrogenase	*M. paratuberculosis*	Q73 × 99	MypaA.01326.g	NAD	3TSC	2.05 Å	Rv0687
Carveol dehydrogenase	*M. thermoresistibile*	E1C9L4	MythA.01326.c	None	3OEC	1.95 Å	Rv0687

**Table 2:** Crystallographic statistics for mycobacterial dehydrogenases.

SSGCID ID	MyabA.01326.f	MyavA.01326.d	MyavA.01326.e	MyavA.01326.g	MyavA.01326.l
Organism	*M. abscessus*	*M. avium*	*M. avium*	*M. avium*	*M. avium*
*Data collection*					
Source	Rigaku FR-E+	CSLI 81D	APS 21 ID-G	Rigaku FR-E+	ALS 5.0.1
Wavelength (Å)	1.5418	0.97949	0.97856	1.5418	0.97946
*Data reduction*					
Space Group	*P*1	*P*2_1_2_1_2_1_	*P*2_1_	*C*222_1_	*C*2
Unit Cell	*a* = 69.36 Å, *b* = 84.97 Å, *c* = 100.89 Å α = 81.77°, β = 76.78°, γ = 74.23°	*a* = 69.55 Å, *b* = 108.41 Å, *c* = 148.30 Å α = β = γ = 90°	*a* = 97.61 Å, *b* = 59.44 Å, *c* = 108.65 Å α = γ = 90°, β = 90.1°	*a* = 82.53 Å, *b* = 106.37 Å, *c* = 135.87 Å α = β = γ = 90°	*a* = 156.47 Å, *b* = 147.51 Å, *c* = 83.24 Å α = γ = 90°, β = 102.49°
Protein copies	8	4	4	2	6
Solvent content (%)	47.9	43.3	53.3	47.8	52.7
V_m_ (Å^3^/Da)	2.36	2.17	2.63	2.36	2.60
Resolution (Å)	50–2.10 (2.15–2.10)^a^	50–1.95 (2.00–1.95)	50–1.55 (1.59–1.55)	50–1.95 (2.00–1.95)	50–2.0 (2.05–2.00)
I/σ	11.1 (5.6)	13.8 (3.8)	12.8 (2.4)	21.8 (2.8)	12.4 (2.5)
Completeness (%)	95.0 (89.3)	96.8 (98.7)	89.3 (93.6)	98.3 (92.0)	98.1 (88.2)
R_merge_	0.105 (0.249)	0.095 (0.480)	0.061 (0.468)	0.052 (0.382)	0.068 (0.369)
Multiplicity	3.9 (3.6)	4.1 (3.7)	2.7 (2.4)	5.8	3.6 (2.8)
Reflections	119,101	79,986	161,212	43,134	121,812
*Refinement*					
R	0.192 (0.198)	0.162 (0.198)	0.146 (0.234)	0.208 (0.322)	0.173 (0.253)
R_free_^b^	0.237 (0.279)	0.195 (0.260)	0.160 (0.247)	0.241 (0.344)	0.214 (0.298)
r.m.s.d. bonds (Å)	0.014	0.012	0.014	0.010	0.015
r.m.s.d. angles (°)	1.484	1.545	1.534	1.441	1.501
Wilson *B*-factors (Å^2^)	18.9	26.6	20.0	32.4	32.7
Mean *B*-factors (Å^2^)	12.3	21.3	14.8	40.8	28.4
*Validation*					
Ramachandran Favored (%)	96.6	96.0	97.1	97.4	95.8
Ramachandran Allowed (%)	99.8	100	100	100	99.9
Molprobity Score	1.47 (98^th^ percentile)	1.39 (98^th^ percentile)	1.06 (99^th^ percentile)	1.29 (99^th^ percentile)	1.30 (99^th^ percentile)
Clash Score	3.02 (99^th^ percentile)	2.51 (99^th^ percentile)	1.55 (99^th^ percentile)	1.47 (100^th^ percentile)	2.34 (99^th^ percentile)
PDB ID	3S55	3T7C	3UVE	4RGB	3PXX

^a^Values in parenthesis indicate the highest resolution shell. 20 shells were used in XSCALE.

^b^A unique 5% of the reflections were used to calculate the R_free_.

**Table 3:** Crystallographic statistics for mycobacterial dehydrogenases.

SSGCID ID	MyavA.01326.q	MypaA.01326.b	MypaA.01326.d	MypaA.01326.g	MythA.01326.c
Organism	*M. avium*	*M. paratuberculosis*	*M. paratuberculosis*	*M. paratuberculosis*	*M. thermoresistible*
*Data collection*					
Source	ALS 5.0.1	ALS 5.0.3	ALS 5.0.3	ALS 5.0.1	Rigaku FR-E+
Wavelength (Å)	0.9774	0.97946	0.9765	0.9774	1.5418
*Data reduction*					
Space Group	*P*222_1_	*C*2	*P*1	*P*2_1_	*C*2
Unit Cell	*a* = 60.79 Å, *b* = 132.14 Å, *c* = 154.94 Å α = β = γ = 90°	*a* = 130.03 Å, *b* = 57.92 Å, *c* = 131.74 Å α = γ = 90°, β = 91.35°	*a* = 64.46 Å, *b* = 70.76 Å, *c* = 125.68 Å α = 97.01°, β = 93.92°, γ = 86.91°	*a* = 57.98 Å, *b* = 127.17 Å, *c* = 69.69 Å α = γ = 90°, β = 104.49°	*a* = 67.86 Å, *b* = 120.57 Å, *c* = 134.56 Å α = γ = 90°, β = 94.05°
Protein copies	4	4	8	4	4
Solvent content (%)	51.8	41.7	51.0	42.4	38.0
V_m_ (Å^3^/Da)	2.55	2.11	2.49	2.13	2.01
Resolution (Å)	50–2.15 (2.20–2.15)^a^	50–1.85 (1.90–1.85)	50–1.5 (1.54–1.50)	50–2.05 (2.10–2.05)	50–1.95 (2.00–1.95)
I/σ	15.2 (3.2)	14.2 (3.6)	21.0 (4.1)	10.5 (3.2)	10.2 (2.9)
Completeness (%)	99.6 (99.9)	97.9 (97.0)	94.8 (90.3)	98.5 (97.3)	98.9 (96.8)
R_merge_	0.110 (0.536)	0.093 (0.471)	0.039 (0.304)	0.130 (0.474)	0.089 (0.316)
Multiplicity	5.8 (5.8)	4.7 (4.6)	3.8 (3.5)	4.2	3.6 (2.2)
Reflections	68,679	82,274	333,445	60,295	77,636
*Refinement*					
R	0.149	0.147 (0.200)	0.141 (0.235)	0.165 (0.243)	0.179 (0.228)
R_free_^b^	0.190	0.195 (0.266)	0.159 (0.262)	0.212 (0.310)	0.233 (0.292)
r.m.s.d. bonds (Å)	0.006	0.015	0.016	0.012	0.017
r.m.s.d. angles (°)	0.834	1.484	1.711	1.495	1.536
Wilson *B*-factors (Å^2^)	22.0	21.4	21.2	21.6	20.9
Mean *B*-factors (Å^2^)	27.2	15.0	11.5	14.8	11.8
*Validation*					
Ramachandran Favored (%)	96.4	98.2	97.3	97.4	97.0
Ramachandran Allowed (%)	100	100	100	100	100
Molprobity Score	1.24 (100^th^ percentile)	1.02 (100^th^ percentile)	0.95 (100^th^ percentile)	1.07 (100^th^ percentile)	1.35 (98^th^ percentile)
Clash Score	2.15 (99^th^ percentile)	2.36 (99^th^ percentile)	1.14 (99^th^ percentile)	1.91 (100^th^ percentile)	3.67 (99^th^ percentile)
PDB ID	5EJ2	3PGX	3SX2	3TSC	3OEC

^a^Values in parenthesis indicate the highest resolution shell. 20 shells were used in XSCALE.

^b^A unique 5% of the reflections were used to calculate the R_free_.

**Table 4:** Comparison of mycobacterial SDR protein and cofactor surface area with other NAD- and NADP-dependent dehydrogenases.

Species	PDB ID	Protein surface area (A^2^)	Cofactor total surface area (A^2^)	Cofactor solvent accessible surface area (A^2^)
*M. abscessus*	3S55	11,341	839	12
*M. avium*	3T7C	12,365	839	21
*M. avium*	3UVE	12,334	834	28
*M. avium*	4RGB	11,338	842	7
*M. avium*	3PXX	11,686	841	14
*M. avium*	5EJ2	11,728	852	26
*M. paratuberculosis*	3PGX	11,168	835	19
*M. paratuberculosis*	3SX2	11,320	842	7
*M. paratuberculosis*	3TSC	11,472	840	26
Average mycobacterial SDRs		11,639	840	18
*Acholeplasma laidlawii*	4NBT	10,890	842	99
*Xanthobacter autotrophicus*	4ITU	10,608	849	64
*Homo sapiens*	4CQM	11,373	837	72
*Helicobactor pylori*	4IIN	10,545	836	160
*Bacillus sp. Sg-1*	4NBU	11,127	840	48
Average NAD-bound dehydrogenases		11,015	841	89
*Staphylococcus aureus*	3SJ7	11,371	928	106
*Listeria monocytogenes*	4JRO	11,057	902	103
*Synechococcus elongatus*	4DMM	10,985	927	64
*Brassica napus*	1EDO	10,757	911	64
*Escherichia coli*	4IIU	11,223	916	74
Average NADP-bound dehydrogenases		11,079	916	82

**Table 5:** STD-NMR binding analysis of compounds with *M. avium* carveol dehydrogenase MyavA.01326.d.

Compound	Description	Spike molecule	%STD (Day 0)	%STD cofactor spike (Day 0)	%STD cofactor spike (Day 7)
NAD	Cofactor	N/A^a^	3.8	N/A	N/A
NADH	Cofactor	N/A	2.6	N/A	N/A
(−)-carveol	Substrate	NAD	6.6	9.9	9.0
(R)-(−)-carvone	Product	NADH	6.2	4.3	3.1
(S)-(+)-carvone	Product	NADH	4.6	3.9	4.0
Tricyclazol	Inhibitor	NAD	11.1	8.2	9.3
Tricyclazol	Inhibitor	NADH	8.3	11.8	9.4
N,N-dimethyl-4-nitosoanaline (NDMA)	Redox partner	NADH	6.2	3.7^b^	17.3^b^
2,6-dichloroindophenol (DCIP)	Redox partner	NAD	19.2	19.7	24.6
2,6-dichloroindophenol (DCIP)	Redox partner	NADH	19.0	9.3^b^	23.8^b^

^a^N/A = not applicable.

^b^Partial conversion of NADH to NAD was observed via changes in chemical shift in these samples. No change in chemical shift was observed for any of the other compounds in these samples.

**Table 6:** STD-NMR binding analysis of compounds with *M. avium* carveol dehydrogenase MyavA.01326.l.

Compound	Description	Spike molecule	%STD (Day 0)	%STD cofactor spike (Day 0)	%STD cofactor spike (Day 7)
NAD	Cofactor	N/A^a^	3.6	N/A	N/A
NADH	Cofactor	N/A	2.1	N/A	N/A
(−)-carveol	Substrate	NAD	10.2	3.9	8.8
(R)-(−)-carvone	Product	NADH	13.5	14.0	14.4
(S)-(+)-carvone	Product	NADH	20.5	21.3	19.7
Tricyclazol	Inhibitor	NAD	17.5	19.9	16.1
Tricyclazol	Inhibitor	NADH	19.0	19.5	17.3
N,N-dimethyl-4-nitosoanaline	Redox partner	NADH	7.5	8.3^b^	14.9^b^
2,6-dichloroindophenol (DCIP)	Redox partner	NAD	11.4	11.5	13.8
2,6-dichloroindophenol (DCIP)	Redox partner	NADH	10.2	3.0^b^	13.0^b^

^a^N/A = not applicable.

^b^Partial conversion of NADH to NAD was observed via changes in chemical shift in these samples. No change in chemical shift was observed for any of the other compounds in these samples.

## References

[b1] Hurley T. D., Bosron W. F., Hamilton J. A. & Amzel L. M. Structure of human beta 1 beta 1 alcohol dehydrogenase: catalytic effects of non-active-site substitutions. Proc Natl Acad Sci USA 88, 8149–8153 (1991). 1896463 10.1073/pnas.88.18.8149 PMC52464

[b2] Thoden J. B. . Structural analysis of UDP-sugar binding to UDP-galactose 4-epimerase from *Escherichia coli*. Biochemistry 36, 6294–6304 (1997). 9174344 10.1021/bi970025j

[b3] Rudolph J., Kim J. & Copley S. D. Multiple turnovers of the nicotino-enzyme PdxB require alpha-keto acids as cosubstrates. Biochemistry 49, 9249–9255 (2010). 20831184 10.1021/bi101291d PMC3295541

[b4] Klinman J. P. & Bonnot F. Intrigues and intricacies of the biosynthetic pathways for the enzymatic quinocofactors: PQQ, TTQ, CTQ, TPQ, and LTQ. Chem Rev 114, 4343–4365 (2014). 24350630 10.1021/cr400475g PMC3999297

[b5] Haft D. H. Bioinformatic evidence for a widely distributed, ribosomally produced electron carrier precursor, its maturation proteins, and its nicotinoprotein redox partners. BMC Genomics 12, 21 (2011). 21223593 10.1186/1471-2164-12-21 PMC3023750

[b6] Bruender N. A. & Bandarian V. The Radical S-Adenosyl-l-methionine Enzyme MftC Catalyzes an Oxidative Decarboxylation of the C-Terminus of the MftA Peptide. Biochemistry 55, 2813–2816 (2016). 27158836 10.1021/acs.biochem.6b00355 PMC5331333

[b7] Khaliullin B. . Mycofactocin biosynthesis: modification of the peptide MftA by the radical S-adenosylmethionine protein MftC. FEBS Lett 590, 2538–2548 (2016). 27312813 10.1002/1873-3468.12249

[b8] van der Werf M. J. . Stereoselective carveol dehydrogenase from Rhodococcus erythropolis DCL14. A novel nicotinoprotein belonging to the short chain dehydrogenase/reductase superfamily. J Biol Chem 274, 26296–26304 (1999). 10473585 10.1074/jbc.274.37.26296

[b9] Myler P. J. . The Seattle Structural Genomics Center for Infectious Disease (SSGCID). Infectious disorders drug targets 9, 493–506 (2009). 19594426 10.2174/187152609789105687 PMC2857597

[b10] Fejzo J. . The SHAPES strategy: an NMR-based approach for lead generation in drug discovery. Chem Biol 6, 755–769 (1999). 10508679 10.1016/s1074-5521(00)80022-8

[b11] Shortridge M. D., Hage D. S., Harbison G. S. & Powers R. Estimating protein-ligand binding affinity using high-throughput screening by NMR. J Comb Chem 10, 948–958 (2008). 18831571 10.1021/cc800122m PMC2631241

[b12] Mayer M. & Meyer B. Group epitope mapping by saturation transfer difference NMR to identify segments of a ligand in direct contact with a protein receptor. J Am Chem Soc 123, 6108–6117 (2001). 11414845 10.1021/ja0100120

[b13] Begley D. W., Moen S. O., Pierce P. G. & Zartler E. R. Saturation transfer difference NMR for fragment screening. Curr Protoc Chem Biol 5, 251–268 (2013). 24391096 10.1002/9780470559277.ch130118

[b14] Dhindwal S. . Biochemical studies and ligand-bound structures of biphenyl dehydrogenase from Pandoraea pnomenusa strain B-356 reveal a basis for broad specificity of the enzyme. J Biol Chem 286, 37011–37022 (2011). 21880718 10.1074/jbc.M111.291013 PMC3196096

[b15] Bryan C. M. . High-throughput protein production and purification at the Seattle Structural Genomics Center for Infectious Disease. *Acta crystallographica*. Section F, Structural biology and crystallization communications 67, 1010–1014 (2011). 10.1107/S1744309111018367 PMC3169394 21904042

[b16] Choi R. . Immobilized metal-affinity chromatography protein-recovery screening is predictive of crystallographic structure success. Acta crystallographica. Section F, Structural biology and crystallization communications 67, 998–1005 (2011). 21904040 10.1107/S1744309111017374 PMC3169392

[b17] Kabsch W. Xds. Acta Crystallogr D Biol Crystallogr 66, 125–132 (2010). 20124692 10.1107/S0907444909047337 PMC2815665

[b18] McCoy A. J. . Phaser crystallographic software. J Appl Crystallogr 40, 658–674 (2007). 19461840 10.1107/S0021889807021206 PMC2483472

[b19] Murshudov G. N., Vagin A. A. & Dodson E. J. Refinement of macromolecular structures by the maximum-likelihood method. Acta Crystallogr D Biol Crystallogr 53, 240–255 (1997). 15299926 10.1107/S0907444996012255

[b20] Adams P. D. . PHENIX: a comprehensive Python-based system for macromolecular structure solution. Acta Crystallogr D Biol Crystallogr 66, 213–221 (2010). 20124702 10.1107/S0907444909052925 PMC2815670

[b21] Emsley P. & Cowtan K. Coot: model-building tools for molecular graphics. Acta Crystallogr D Biol Crystallogr 60, 2126–2132 (2004). 15572765 10.1107/S0907444904019158

[b22] Chen V. B. . MolProbity: all-atom structure validation for macromolecular crystallography. Acta Crystallogr D Biol Crystallogr 66, 12–21 (2010). 20057044 10.1107/S0907444909042073 PMC2803126

[b23] Dutta D., Bhattacharyya S. & Das A. K. Crystal structure and fluorescence studies reveal the role of helical dimeric interface of staphylococcal FabG1 in positive cooperativity for NADPH. Proteins 80, 1250–1257 (2012). 22275129 10.1002/prot.24024

[b24] Javidpour P. . Biochemical and structural studies of NADH-dependent FabG used to increase the bacterial production of fatty acids under anaerobic conditions. Appl Environ Microbiol 80, 497–505 (2014). 24212572 10.1128/AEM.03194-13 PMC3911115

